# High-density genetic map construction and quantitative trait loci analysis of the stony hard phenotype in peach based on restriction-site associated DNA sequencing

**DOI:** 10.1186/s12864-018-4952-y

**Published:** 2018-08-14

**Authors:** Shaolei Guo, Shahid Iqbal, Ruijuan Ma, Juan Song, Mingliang Yu, Zhihong Gao

**Affiliations:** 1 College of Horticulture, Nanjing Agricultural University, Nanjing, 210095 China; 2 Institute of Pomology, Jiangsu Academy of Agricultural Sciences, Nanjing, 210014 China; 3 Jiangsu Key Laboratory of Horticultural Crop Genetic Improvement, Nanjing, 210014 China

**Keywords:** Peach, Stony hard, Restriction-site associated DNA sequencing, Genetic map, Quantitative trait loci, Gene expression

## Abstract

**Background:**

Peach (*Prunus persica*) is an important fruit crop that generally softens rapidly after harvest resulting in a short shelf-life. By contrast, stony hard (SH) peach fruit does not soften and hardly produces ethylene. To explore the candidate genes responsible for the SH phenotype, a high-density genetic map was constructed by restriction-site associated DNA sequencing technology.

**Results:**

In the present study, the linkage map consisted of 1310 single nucleotide polymorphism markers, spanning 454.2 cM, with an average marker distance of 0.347 cM. The single nucleotide polymorphisms were able to anchor eight linkage groups to their corresponding chromosomes. Based on this high-density integrated peach linkage map and two years of fruit phenotyping, two potential quantitative trait loci for the SH trait were identified and positioned on the genetic map. Additionally, *Prupe.6G150900.1*, a key gene in abscisic acid (ABA) biosynthesis, displayed a differential expression profile identical to the ABA accumulation pattern: mRNA transcripts were maintained at a high level during storage of SH peaches but occurred at low levels in melting fruit.

**Conclusion:**

Thus *Prupe.6G150900.1* might play a crucial role in the SH phenotype of peach in which ABA negatively regulates ethylene production. Also, this high-density linkage map of peach will contribute to the mapping of important fruit traits and quantitative trait loci identification.

**Electronic supplementary material:**

The online version of this article (10.1186/s12864-018-4952-y) contains supplementary material, which is available to authorized users.

## Background

Peach [*Prunus persica* (L.) Batsch] is a typical climacteric fruit, and it generally softens rapidly after harvest, resulting in a short shelf-life that unfavorably affects its market value [1, 2]. Therefore, peach fruit texture is an important quality in the breeding of fresh market varieties.

To date, peach cultivars have been classified into three flesh texture types, melting (M), no-melting (NM) and stony hard (SH) based on the characteristics of fruit firmness and texture changes during peach ripening and softening [3–5]. Generally, M fruit are characterized by their prominent softening after harvest, NM fruits are characterized by slow softening at the later stages of ripening and never melt, while the SH type does not produce ethylene and barely softens after harvest (both on- and off-tree) [3, 4, 6]. A genetic analysis indicated that the SH genotype is controlled by a single recessive gene [5] and is epistatic to the locus [4].

In general, the softening of climacteric fruit, is related to endo-polygalacturonase enzyme activity, and is induced by ethylene [7–9]. A low level of ethylene production may contribute to the suppression of fruit softening in SH peach cultivars, and this depends on the suppressed transcription of *PpACS1* [10]. The suppression of *PpACS1* is caused by low indole-3-acetic acid concentrations in SH peaches [11]. *PpYUC11* may play an essential role in auxin biosynthesis during peach fruit ripening and is a candidate gene for controlling the SH phenotype in peach [6]. However, Dong et al. [12] showed that ABI4-mediated the transcriptional repression of the ethylene biosynthesis genes *ACS4* and *ACS8* in *Arabidopsis*. Li et al. [13] suggested that in ethylene over-producer mutants, abscisic acid (ABA) treatments suppress *ACS5* transcripts levels and the ethylene content, indicating that ethylene production is determined by various metabolic pathways.

The advancements in next-generation sequencing (NGS) technologies coupled with continually reducing costs, offers unprecedented conditions for genome-wide marker development and genotyping by sequencing [14]. Restriction-site associated DNA sequencing (RAD-seq) is a foremost NGS technology for high-throughput genotyping [15], in which a reduced representation of the genome produced by the DNA flanking specific a restriction enzyme is sequenced, and the reduced genome is bound to an adapter containing multiplex identifiers to form reduced-representation libraries [15–17]. To date, high-density genetic maps of many species, including eggplant [18], ryegrass [19], barley [20], grape [16], sesame [17], pear [21], apple [22] and cowpea [15], have been constructed using the RAD-seq method. However, RAD-seq technology is barely applied in peach.

To clarify the mechanism responsible for the SH phenotype and improve fruit quality, especially texture by peach breeding, further research is needed. Currently, genetic maps constructed with molecular markers have been constructed to detect the genomic loci or genes related to the traits of interest may provide efficient methods [23]. To explore the candidate gene which controls the SH phenotype, the phenotype-related quantitative trait loci (QTLs) were identified through a high-density single nucleotide polymorphism (SNP) linkage map of the peach genome with the RAD-seq technology and a family of 103 F_1_ lines phenotypic identification.

## Results

### Phenotypic identification

**Fig. 1:**
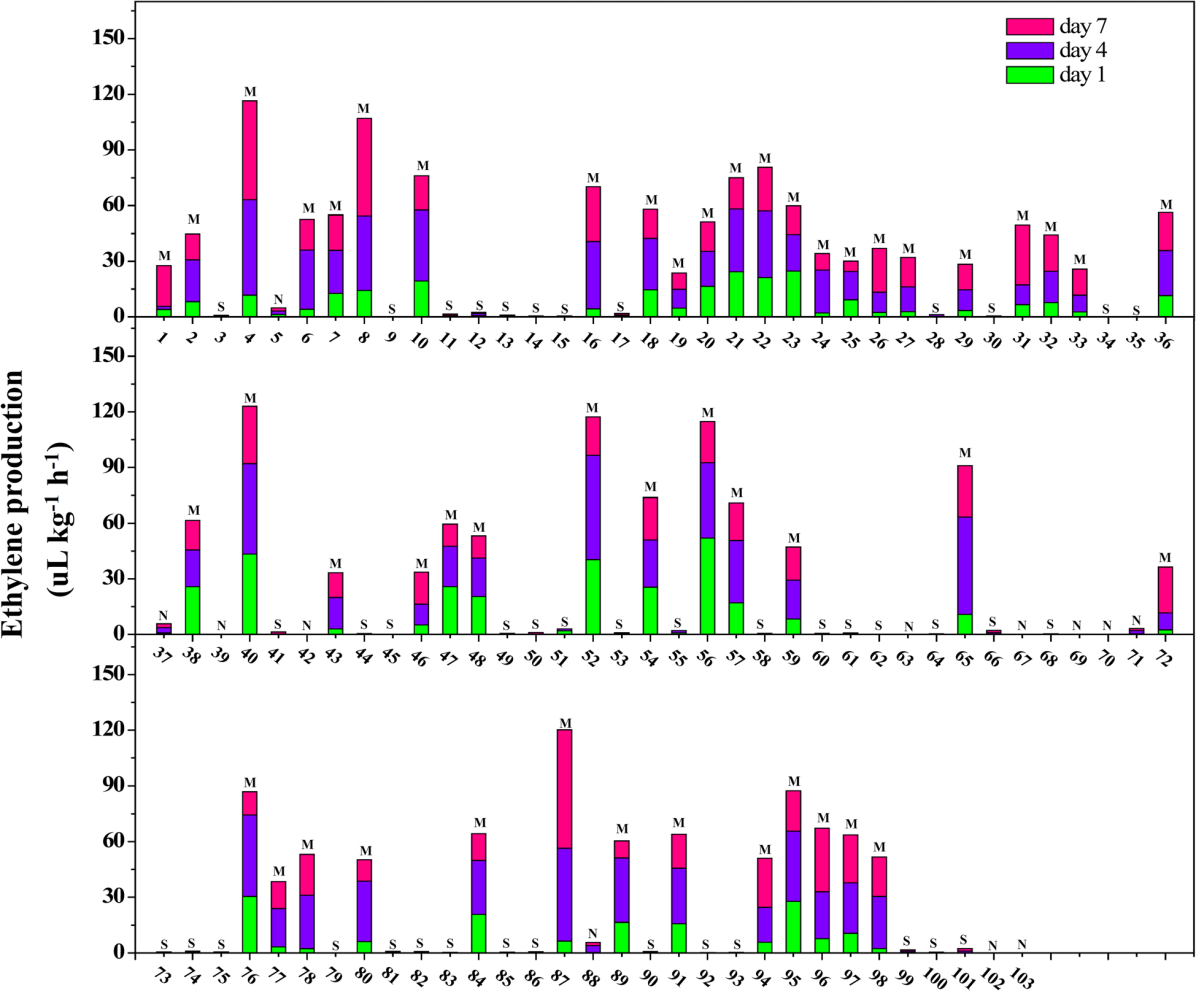
Ethylene production in F_1_ offspring during storage at room temperature (2016). Numbers on the *x*-axis represent individuals; the *y*-axis represents ethylene production; different colors represent the days after harvest when samples were measured; The letter ‘S’, ‘M’ and ‘N’ represent stony hard phenotype, melting phenotype and unidentified phenotype, respectively. Data are means ± SEs (*n* = 3)

**Fig. 2:**
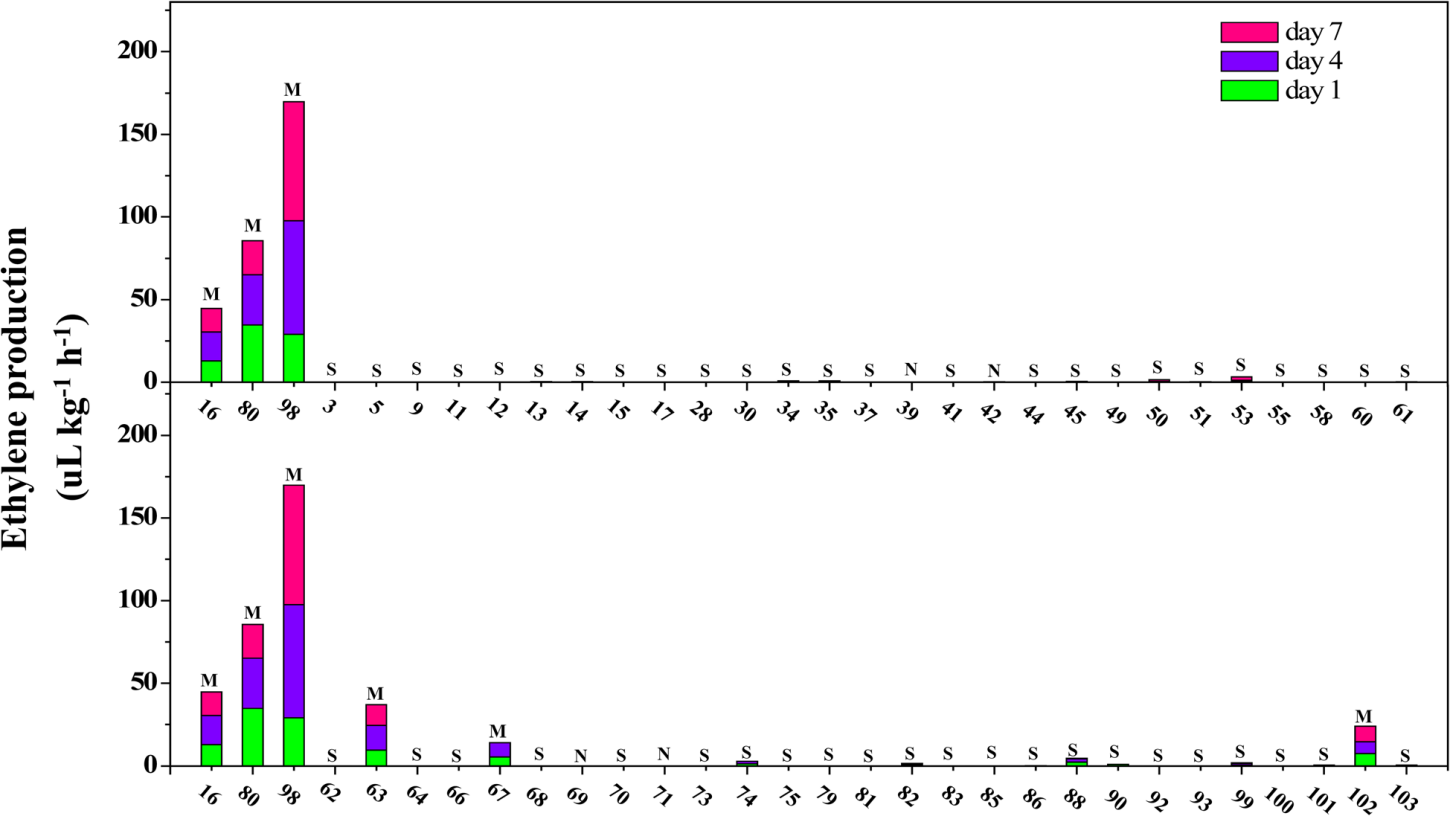
Ethylene production in F_1_ offspring during storage at room temperature (2017). Numbers on the *x*-axis represent individuals; the *y*-axis represents ethylene production; different colors represent the days after harvest when samples were measured; The letter ‘S’, ‘M’ and ‘N’ represent stony hard phenotype, melting phenotype and unidentified phenotype, respectively. Individuals of 16, 80 and 98 as a contrast. Data are means SEs (n = 3)

**Fig. 3:**
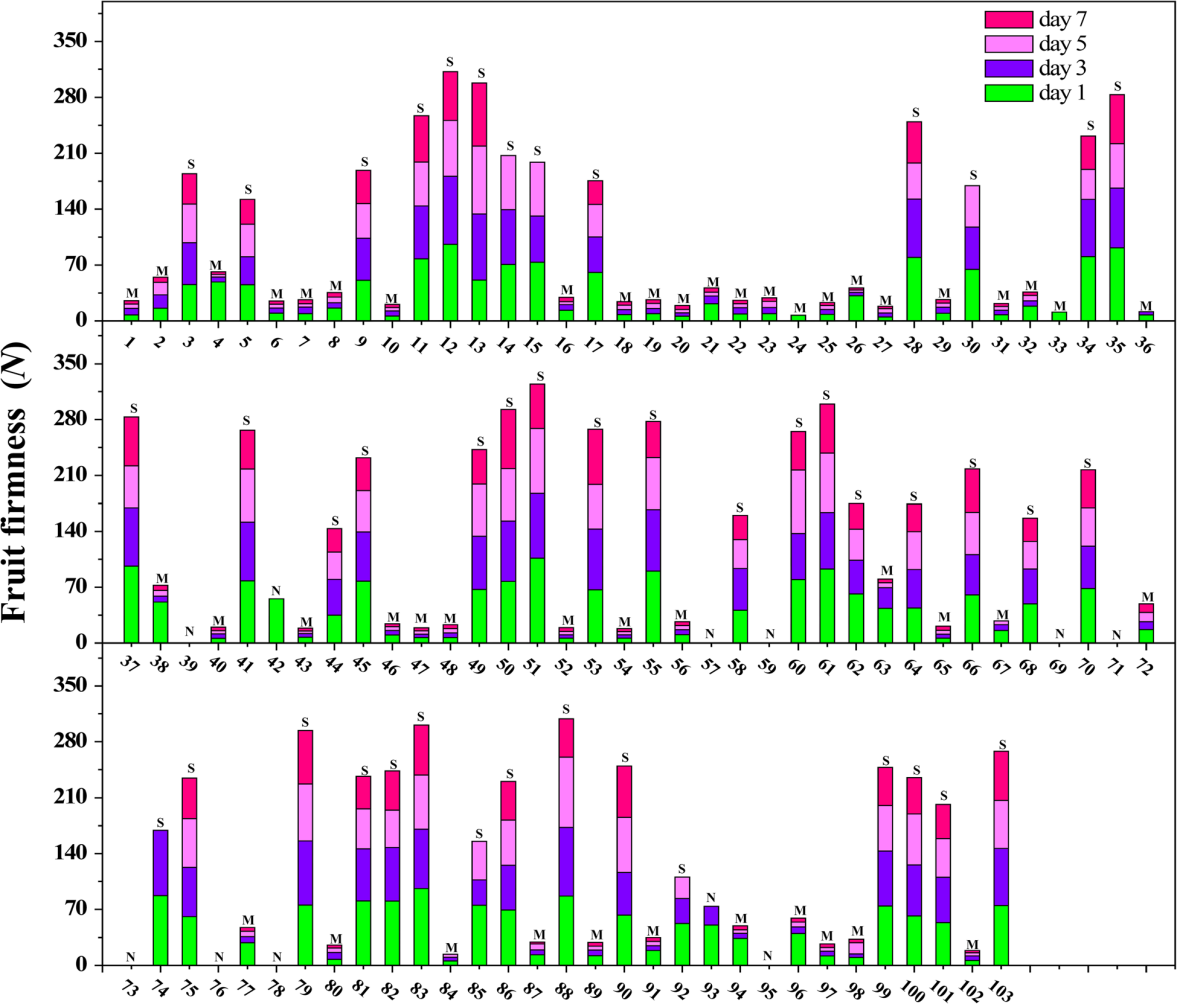
Fruit firmness of F_1_ offspring during storage at room temperature (2017). Numbers on the *x*-axis represent individuals; the *y*-axis represents fruit firmness; different colors represent the days after harvest when samples were measured; The letter ‘S’, ‘M’ and ‘N’ represent stony hard phenotype, melting phenotype and unidentified phenotype, respectively. Data are means ± SEs (n = 3)

In the present study, we identified 42 SH and 49 M fruit trees based on the ethylene production phenotype during storage at room temperature in 2016, and individuals 5, 37, 39, 42, 63, 67, 69, 70, 71, 88, 102 and 103 were unidentified due to less fruits (Fig. 1 and Additional file 1a). In addition, all 42 SH fruit tree and 12 unidentified trees were identified according to ethylene production in 2017. The 42 SH fruit trees identified in 2016 also exhibited the SH phenotype in 2017 (hardly synthesizing ethylene), and trees 5, 37, 70, 88 and 103 were identified as SH phenotype in 2017 according to ethylene production. Trees 63, 67 and 102 were identified as M phenotype, while trees 39, 42, 69 and 71 remained unidentified owing to less fruits in 2017 (Fig. 2). Furthermore, 45 fruit trees were identified as SH phenotype and 47 fruit trees as M phenotype based on the phenotype of firmness in 2017, while 11 fruit trees remained unidentified (39, 42, 57, 59, 69, 71, 73, 76, 78, 93 and 95) owning to less fruits (Fig. 3 and Additional file 1b). The segregation of the SH phenotype in the F_1_ family (103) of the cross between ‘Yumyeoung’ (YM) (female) and ‘Hujingmilu’ (HJML) (male) presented 1:1 ratio (Table 1) based on both ethylene production and fruit firmness, which indicated that the SH phenotype may be controlled by a single recessive gene. Finally, the phenotypic identifications based on ethylene production in 2016 and 2017 were used for a QTL analysis (Additional file 1c).

**Table 1:** Segregation of the SH phenotype in F_1_ population (103) of the cross between ‘YM’ and ‘HJML’

Year	based trait	SH^a^	M^b^	Unidentified	Expected rate	χ^2c^
2016	ethylene	42	49	12	1:1	1.650
2017	firmness	45	47	11	1:1	1.010

^a^SH, stony hard

^b^M, melting

^c^χ^2^ represents the chi-squared value with the *P*-value set at 0.05

### ABA content determination

**Fig. 4:**
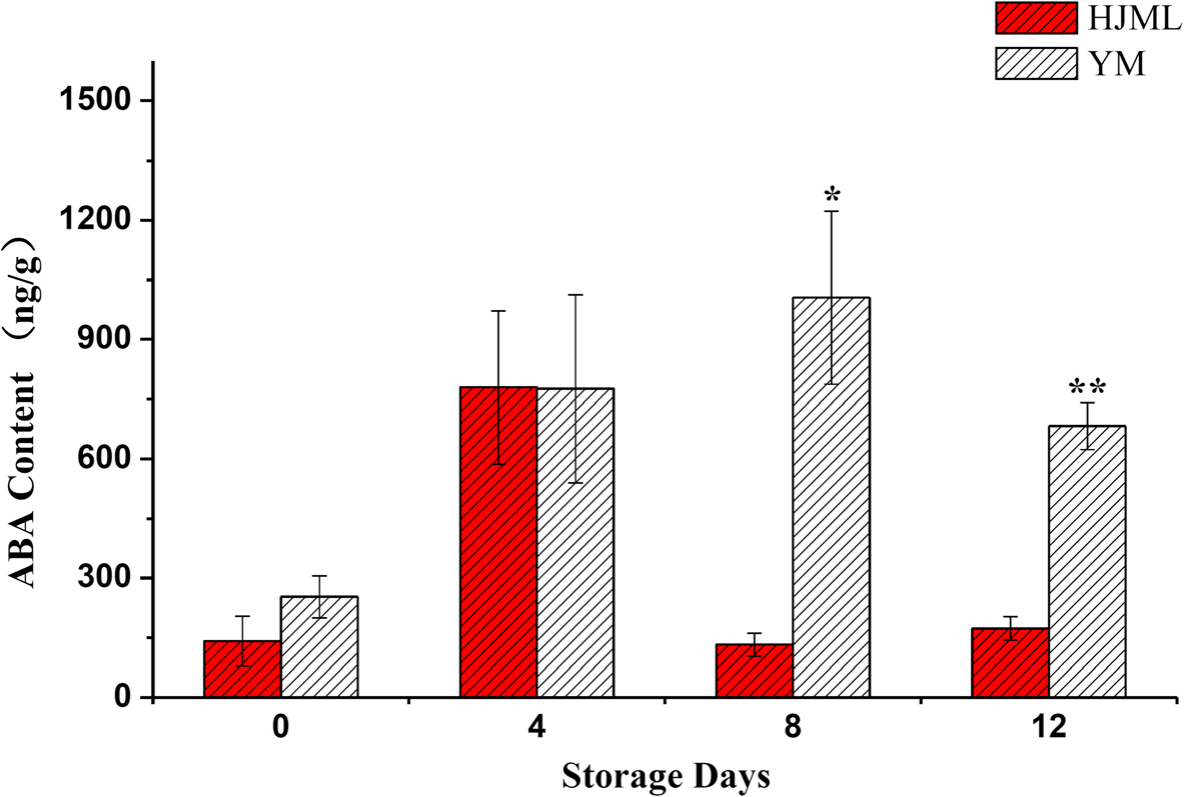
ABA contents of ‘HJML’ and ‘YM’ during storage at room temperature. The *x*-axis represents the storage days after harvest; the *y*-axis represents the ABA content. Data are means ± SEs (n = 3). Significant differences (*p* < 0.05) between means are indicated by the symbol ‘*’ and significant differences (*p* < 0.01) between means are indicated by the symbol ‘**’

There is no significant difference in the early stage of storage between the SH fruit ‘YM’ and the M fruit of ‘HJML’ of the ABA content, but significantly higher in ‘YM’ cultivar than that in ‘HJML’ cultivar during the later storage period (Fig. 4).

### RAD-seq and linkage map construction

**Fig. 5:**
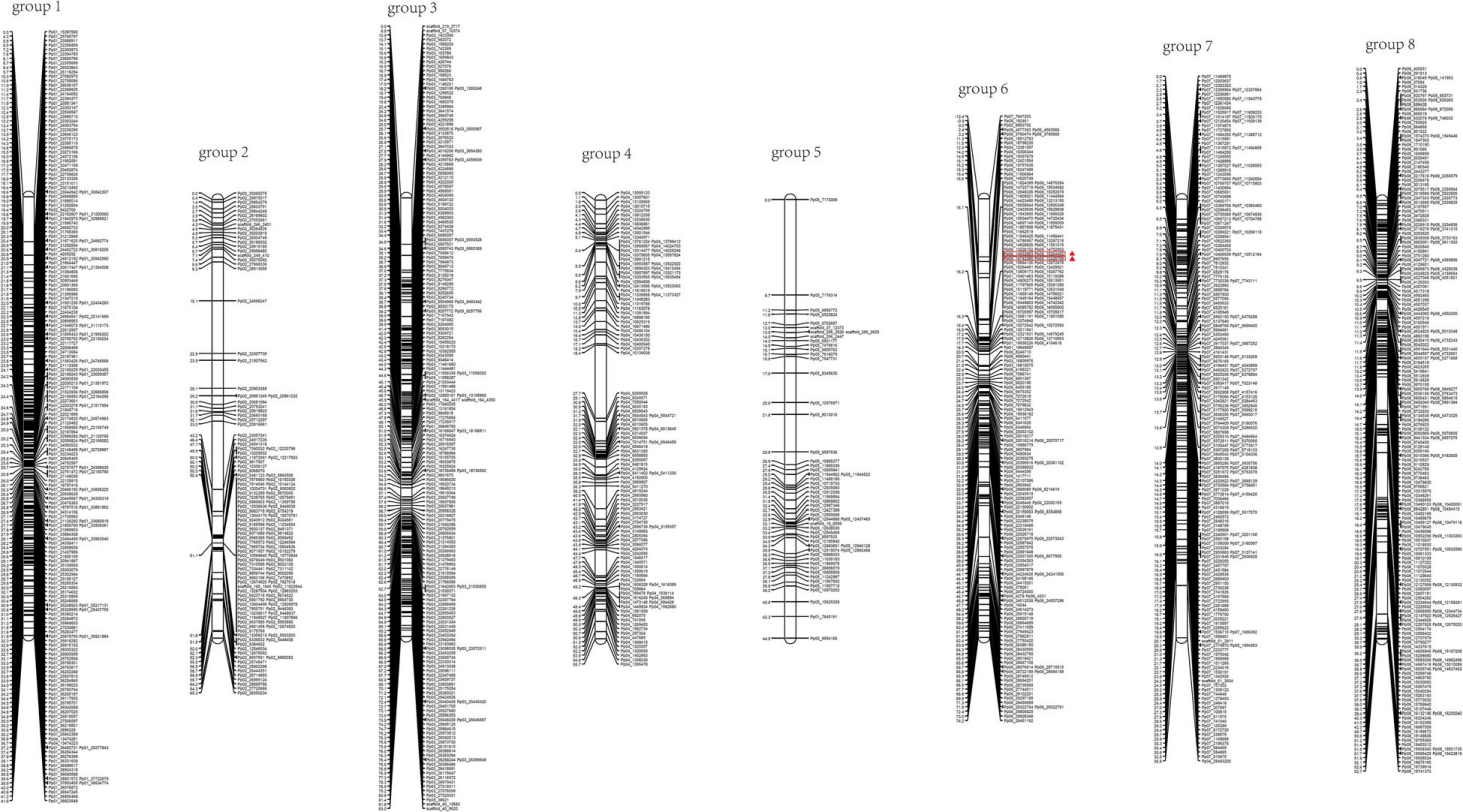
Integrated linkage groups 1 to 8 for ‘YM’ × ‘HJML’. Marker names are shown to the right of each group, and map distances (in cM) are shown on the left. Red symbols represent the position of QTLs

After filtering, there were 8550.55 million clean reads, consisting of ∼85.51 Gb, that were used to generate RAD tags (Additional file 2). Thus, the reads ensured that more than 91 and 96% of the nucleotides had a quality values above Q30 (equal to a 0.1% sequencing error) and Q20 (equal to a 1% sequencing error), respectively (Additional file 2). The GC content for both parents and offspring were ∼38.7%. A total of 6150 SNP markers were used for linkage map construction (Additional file 3). As a result, 1310 markers were selected and mapped onto eight different linkage groups (LGs), covering 454.2 cM of the peach genome and having an average distance of 0.347 cM between adjacent markers (Table 2 and Fig. 5). The length of individual LGs ranged from 36.9 cM (LG7) to 83.0 cM (LG3), with inter-locus distances of between 0.18 (LG7) and 0.809 cM (LG5). LG1 was the densest, having 212 SNP loci, while LG5 had the least number of SNP loci (55). A linkage map including genetic distances and loci names, associated with SNP marker positions in different LGs is presented in Fig. 5.

**Table 2:** Distribution of mapped markers among eight linkage groups in peach

Linkage group	Number of markers	Length (cM)	Average distance (cM)
LG1	212	41.9	0.198
LG2	129	65.3	0.506
LG3	195	83.0	0.426
LG4	119	55.7	0.468
LG5	55	44.5	0.809
LG6	186	74.2	0.399
LG7	205	36.9	0.180
LG8	209	52.7	0.252
Total	1310	454.2	
Average	163.75	56.775	0.347

### SNP and QTL analyses

Overall, 51,253 polymorphic SNPs were identified between the two parental genotypes and those of their individual offspring (Additional file 4). To characterize the SNPs in peach, we determined the percentages of different SNP types. Among these SNPs, the dominant types were transitions, and the A/G and C/T types represented to 32.1 and 31.9% of the SNPs, respectively. The other four SNP types were the trans-versions A/T, A/C, G/T, and G/C. Their proportions varied from 6.9 to 10.0%, accounting for 36% of all SNPs (Table 3). All of the SNP types present in each individual are summarized in Additional file 5. A High-Density Genetic Map constructed using two years of fruit phenotyping was employed to identify SH phenotype-linked QTL in peach. Two SH phenotypic-related QTLs were found in one linkage group (LG6) using the ICIM-ADD method (Table 4).

**Table 3:** Statistics of all SNP types in the parent plants and their offspring

SNP types	Numbers	Ratio (%)
A/T	274, 225	10.0
A/G	879, 057	32.1
A/C	256, 293	9.4
G/T	265, 966	9.7
G/C	188, 305	6.9
C/T	874, 086	31.9
Total SNP numbers	273, 793, 2

**Table 4:** Analysis of SH phenotypic-related QTLs in peach

Trait name	Group	Left marker	Right marker	LOD^a^	PVE(%)^b^
SH	6	Pp06_14932683	Pp06_15264452	9.2038	44.19%
SH	6	Pp06_14742390	Pp06_12326708	2.5097	42.29%

^a^LOD, likelihood of odds

^b^PVE, phenotypic variation explained

### Functional analysis of candidate genes

**Fig. 6:**
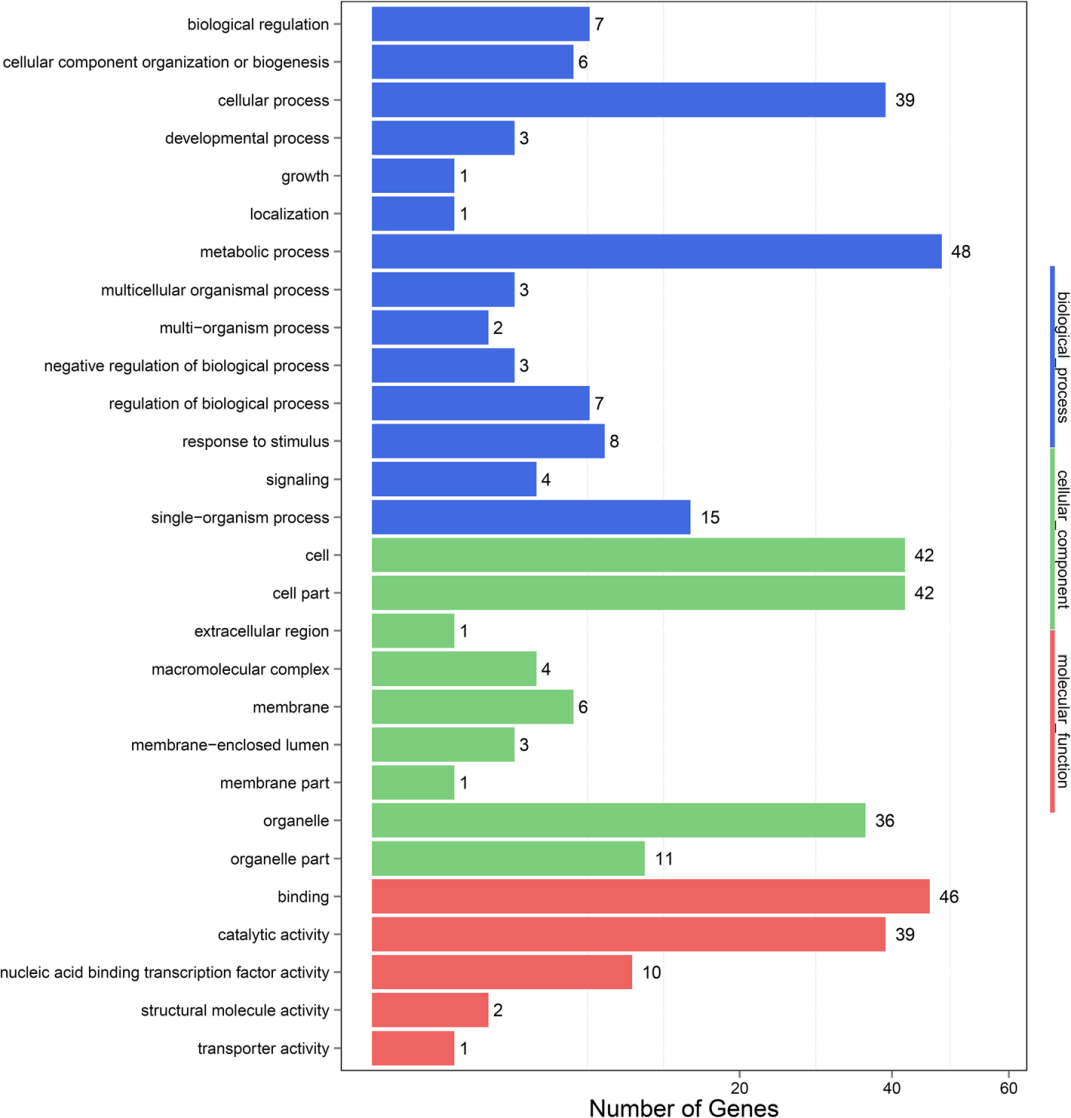
Histogram showing the gene ontology functional enrichment of differentially expressed genes. Different colors represent distinct functional groups

A total of 249 discrete genes were obtained from the two QTL regions (Additional file 6). Of these 80 genes were identified in the Gene Ontology (GO) database and, could be categorized into 28 functional groups, including 14 in biological processes, 9 in cellular components and 5 in molecular functions. The distribution of these eighty genes ranged from 1(in growth) to 48 (in metabolic processes) in the different functional groups. Most of the genes were involved in the cellular process (39), metabolic process (48), cell (42), cell part (42), binding (46) and catalytic activity (39) (Fig. 6). The distribution of the 80 genes in GO Database is list in Additional file 6. Of the 249 candidate genes, 84 were identified in the Kyoto Encyclopedia of Genes and Genomes (KEGG) pathway database, and they were associated with 19 pathways. The pathway with the highest enrichment factor was carotenoid biosynthesis, and a Q-value of < 0.05 was found for plant hormone signal transduction, carotenoid biosynthesis, phenylpropanoid biosynthesis, cyanoamino acid metabolism, starch and sucrose metabolism and regulation of autophagy (Additional file 7). These pathways may be significantly involve in determining the SH phenotype, and the 84 genes identified in the KEGG pathway database are listed in Additional file 6.

### Expression analysis using real-time quantitative PCR (RT-qPCR)

**Fig. 7:**
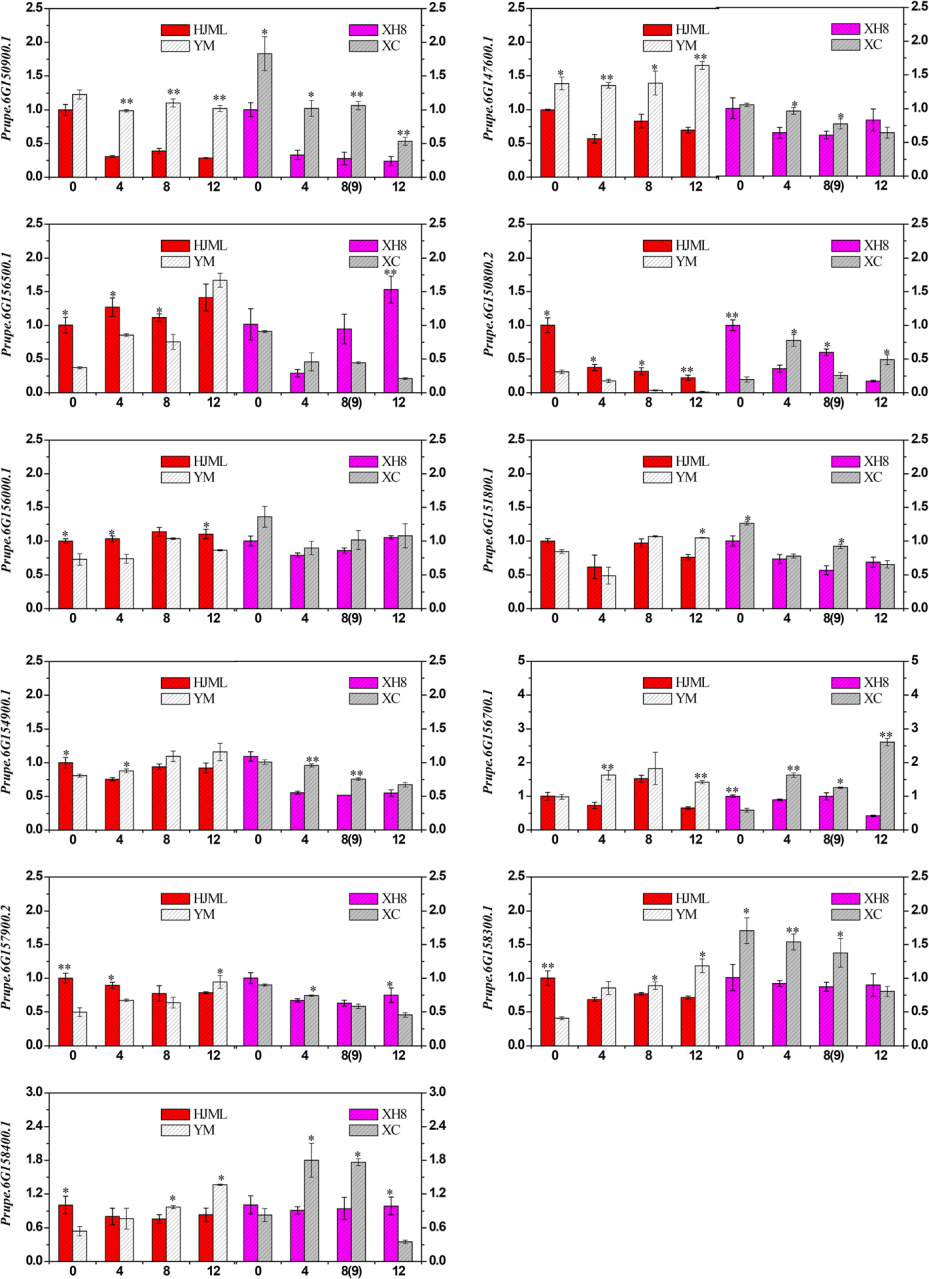
Relative mRNA expression levels of candidate genes during fruit softening. The *x*-axis represents the storage days after harvest; the *y*-axis represents relative expression levels of genes. Data are means ± SEs (*n* = 3). Significant differences (*p* < 0.05) between means are indicated by the symbol ‘*’ and significant differences (*p* < 0.01) between means are indicated by the symbol ‘**’

249 genes were aligned to the Genome Database for *Rosaceae* (GDR) (https://www.rosaceae.org/node/1) to identify the candidate genes. In total, 11 candidate genes (with *P*-value < 0.05 in pathway) in peach cultivars with distinct softening characteristics were analyzed using RT-qPCR (Fig. 7). In the present study, the expression of *Prupe.6G150900.1* was significantly higher in SH fruit (‘YM’) than that in M fruit (‘HJML’) during storage (Fig. 7), the same phenomenon occurred in other M (‘XH8’) and SH (‘XC’) fruit, which indicated that *Prupe.6G150900.1* was expressed higher in SH fruit than in M fruit. The profiles were similar to those of the ABA content in ‘HJML’ and ‘YM’. Likewise, *Prupe.6G147600.1* was expressed significantly higher in ‘YM’ than in ‘HJML’ fruit, but in ‘XH8’ and ‘XC’ expressed no significant diversity during later storage stage. *Prupe.6G156500.1* was expressed significantly higher in ‘HJM’L fruit than in ‘YM’ fruit (Fig. 7) and also expressed no significant diversity in ‘XH8’ and ‘XC’ cultivars. The other genes in Fig. 7 showed no significant diversity between SH and M-type cultivars.

## Discussion

### SH inheritance in peach

SH phenotype is projected to be used as a genetic resource for breeding to meet fresh market requirements [24]. Additionally, identifying the mechanisms responsible for the SH phenotype is of great importance. The segregation of the SH phenotype in the F_1_ family (103) of the cross between ‘YM’ and ‘HJML’ in our study was consistent with Mendelian inheritance (1,1ratio). Which suggested that the SH trait is controlled by a single recessive gene. These results are consistent with Yoshida’s research [5].

### Construction of a high-density genetic map for peach

Generally, molecular markers and genetic maps are widely used in the genetic mapping of important traits in crops [25]. RAD-seq technology has been employed as an efficient tool for high-density genetic mapping and QTL analyses with the capacity to discover larger numbers of markers in any organism (with or without reference genomes) [15, 26–29]. To date, no RAD-seq-based SNP discover technique has been reported in peach. In the present study, a high-density SNP linkage map in peach was constructed and characterized using the RAD-seq method. In 1994, Chaparro et al. [30] constructed the first linkage map of peach. With the development of molecular marker technology, RFLP, RAPD, AFLP and SSR markers [31–36], especially SSR were widely used to construct linkage maps in peach. Compared with microsatellites and other markers, SNPs are efficient tools for linkage mapping that can be genotyped on a more abundant and much larger scale [21, 37]. The ‘Texas’ × ‘Earlygold’ genetic map is considered the reference map for *Prunus,* having 562 markers and, covering 517 cM of the peach genome with an average distance of 0.92 cM between adjacent markers (http://www.rosaceae.org/maps) [38]. Recently, two high-density linkage maps were constructed: the Pop-DF map, covering 422 cM of the peach genome and including 1037 SNP markers (0.407 cM/SNP locus), and the Pop-DG map covering 369 cM and including 738 SNPs (0.5 cM/SNP locus) [39]. The map size reported here is 454.2 cM, which is shorter than the previously published maps of 562.3 cM [35] and 1061.8 cM [40] in peach. This might be the result of the limited LGs linkage groups or segregation distortion markers present in our map. The same phenomenon occurred for sesame [17]. This was the first mapping family reported in peach. Additionally, this genetic map had the highest marker density, and fewer distortion markers compared with other published genetic maps in peach [35, 38, 41]. The inter-marker distance of 0.347 cM suggested that it would be favorable for locating sequence scaffolds on the physical peach genome sequence map. Because the whole-genome sequence of peach has been released [42], it could be beneficial for obtaining genes near each marker.

### Identification of QTLs related to the SH phenotype in peach

Mapping QTLs in peach is challenging because the peach plant is highly heterozygous with a long growth and breeding cycle. Mapping and QTL have been performed using F_1_ [39, 41, 43] F_2_ and BC1 families, as shown at http://www.rosaceae.org/maps. Fruit quality is considered the primary significant selection criteria by peach breeders [44] and, mainly includes color, flavor and texture. Shen et al. [35] mapped the Dominant Blood-Flesh locus, which may control the blood-flesh phenotype of ‘Wu Yue Xian’ peach to the top of LG5. Aromatic and other volatile compounds were mapped onto the *Prunus* reference map using gas chromatography-mass spectrometry [45, 46]. The NM flesh trait locus may be located in the central region of LG4 with recessive alleles determining the characteristics [47]. The activity of an endo-polygalacturonase gene was associated with M flesh [48]. Recently, Serra et al. [49] identified the qP-MD5 QTL as a key factor for slow M flesh, and qP-MD6 may modulate the maturity date trait. With the development of sequencing and SNP-genotyping platforms, high-resolution linkage maps have been successfully used to locate qualitative and quantitative traits [21, 50]. A total of 32 potential QTLs for 11 traits (5 quantitative and 6 qualitative traits), including length of pedicel, single fruit weight, soluble solid content, transverse diameter, vertical diameter, calyx status, flesh color juice content, number of seeds, skin color, and skin smooth, were identified and positioned in pear [21]. As an important texture type, the SH phenotype is of importance in peach [4, 6, 10, 11]. In this study, the pathway with the highest enrichment factor was carotenoid biosynthesis and plant hormone signal transduction, these indicated that the SH phenotype may much correlation with ABA, and the crosstalk of ethylene and ABA is important for multiple physiological processes [51–53]. These will be of great beneficial in clarifying the mechanism responsible for the SH phenotype.

### Identification and expression analyses of candidate genes

Three genes (*Prupe.6G150900.1*, *Prupe.6G147600.1* and *Prupe.6G156500.1*) were identified as good candidates for controlling the SH trait. *Prupe.6G150900.1* was annotated as encoding abscisic-aldehyde oxidase 3, which was thought to catalyze the oxidation of the abscisic aldehyde reaction in the last step of ABA biosynthesis [54]. The expression of *Prupe.6G150900.1* in the SH-type cultivar (‘YM’) was significantly higher than that in the M-type cultivar (‘HJML’) during storage. The same expression phenomenon also occurred in the ‘XC’ (SH) and ‘XH8’ (M) cultivars. Furthermore, the ABA content in the SH-type cultivar (‘YM’) was significant high than in M-type cultivar (‘HJML’). Thus the SH phenotype may have a close relationship with ABA. Multiple physiological processes are determined by the crosstalk between ABA and ethylene [51–53]. Particularly, the induction of ethylene biosynthesis can be prevented by ABA treatments [53, 55, 56]. Recent research suggested that ABA negatively regulates ethylene production through the ABI4-mediated transcriptional repression of the ethylene biosynthesis genes *ACS4* and *ACS8* in *Arabidopsis* [12]. This indicates that *Prupe.6G150900.1* may be an important factor in the inhibition of ethylene production through ABA. In addition *Prupe.6G147600.1* was annotate as being related to the transcription factor YABBY, and *Prupe.6G156500.1* was annotate as encoding beta-glucosidase (EC3.2.1.21) an important hydrolase that may be related to the softening of peach fruit. In M and NM fruit cultivars, the endo-polygalacturonase activity during peach fruit ripening is responsible for the difference in softening [8, 9]. Ethylene treatment resulted in SH type peach fruit softening rapidly and increasing endo-polygalacturonase enzyme activity and PpPG mRNA expression [57, 58]. And *Prupe.6G156500.1* may related to the fruit softening as endo-polygalacturonase for the higher expression in M cultivars and lower expression in SH cultivars. Further studies are needed to reveal the candidate genes’ function in peach.

## Conclusion

The use of high-density genetic map will be beneficial for mapping important fruit traits and for QTL identification in peach. *Prupe.6G150900.1* may be an important factor involved in the inhibition of the ethylene production through ABA and might be an important candidate gene for controlling the SH phenotype. These results will be useful for further analyses of SH phenotype.

## Methods

### Materials and DNA extraction

The two parents showed distinct softening characteristics, including fruit firmness and ethylene production. ‘YM’ and ‘HJML’ have significantly different texture phenotypes: ‘YM’ is SH, maintaining fruit firmness and hardly synthesizing ethylene during storage at room temperature, and ‘HJML’ is M, rapidly softening and exhibiting an ethylene production peak during storage at room temperature [59]. The sample family consisted of 103 F_1_ progeny from a cross between two peach cultivars: the SH-type cultivar ‘YM’ and the M-type cultivar ‘HJML’. The family was hybridized in 2008, These plants were grown at the experimental peach orchard in Nanjing, Jiangsu, China. Young leaves (first few leaves of the apex) were collected and immediately stored in liquid nitrogen and transferred to a − 80 °C freezer in the laboratory. A sample from each individual was ground in liquid nitrogen and total genomic DNA was extracted using a Plant Genomic DNA Kit (TIANGEN, Beijing, China). A 1% agarose gels and a spectrophotometer (Qubit 2.0 Fluorometer, Invitrogen) were used to determine the genomic DNA’s integrity and quality, respectively.

### Detection of ethylene production and fruit firmness

Ethylene production was measured by gas chromatography (Agilent 7890A, CA, USA) according to the method followed by Guo et al. [59] in 2016 and 2017, and fruit flesh firmness was measured using the TA-XT. Plus Texture Analyser (Stable Micro Systems Ltd., Godalming, Surrey, UK) according to the method of Guo et al. [59] in 2017. All fruit samples reached commercial maturity, had no diseases and mechanical damage with uniform maturity were randomly collected. And the fruits were stored in a room at at 25 ± 0.5 °C with a relative humidity of 75–85%. Ten fruits are collected each time. Three independent biological replicates were conducted for these measurements.

### Determination of the ABA content

The ABA content was measured using ESI-HPLC-MS/MS (Waters, Milford, USA) as described previously [60]. Briefly, 0.6 g of peach flesh was homogenized in liquid nitrogen, transferred to a 20-mL centrifuge tube with 5 mL of isopropanol/hydrochloric acid extraction buffer, and then the reaction solution was vortexed for 30 min at 4 °C. Subsequently, 10 mL dichloromethane was added, and the mixture was vortexed at 4 °C for 30 min. Then, samples were centrifuged at 13000 rpm for 5 min to separate the organic phase, which was subsequently protected from light and dried under nitrogen gas. It was then re-dissolving dissolved 400 μL methanol/methane acid (99.9/0.1, *v*/v). Then, the solution was sequentially passed through a 0.22 μm filter membrane to measure with HPLC-MS/MS. The HPLC separation used an ACQUITY UPLC R BEH C18, 100 mm × 2.1 mm × 1.7 μm column (Waters) and was eluted with H_2_O/methanol (98/2, v/v), 0.05% methane acid and 5 mmol/L ammonium acetate (eluent A) and acetonitrile (eluent B) at a flow rate 0.3 mL min^− 1^. The temperature of the column was maintained at 40 °C and the sample size was 5 μL. The MS methods were as follows: positive, negative ion electrospray ionization, capillary voltage of 3.0 Kv, ionization temperature of 150 °C, cone gas flow of 50 L/Hr, de-solvation temperature of 400 °C, de-solvation gas flow of 800 L/Hr, monitoring mode of MRM. Comparisons of the peak area ratio (analyte/IS) to concentrations were used to construct the calibration curve, and then the ABA content was calculated based on the calibration curve. Three biological replicates were conducted.

### RAD library construction and sequencing

Illumina DNA sequencing combined with a RAD strategy was used for the effective identification of SNP markers. The RAD-seq library construction protocol was similar to that described in a set of previously published papers [21, 26] . Briefly, 1 μg genomic DNA from each individual was digested by the restriction enzyme *Eco*R1(NEB), and the P1 adapter was added with T4 DNA ligase (NEB) for 1 h at 37 °C. Ligation products were pooled and fragmented by a Covaris sonicator. Then fragments between 300 and 500 bp were excised after agarose gel electrophoresis selection and purified using a QIAquick Gel Purification Kit (Qiagen). The purified products were combined with End Repair Mix incubated at 20 °C for 30 min, and then purified again. An end-repaired DNAdA overhang was added by the A-Tailing mix (NEB) at 37 °C for 30 min. The P2 adapter was added to the product for 15 min at 20 °C, and the samples were then purified using a QIAquick Gel Purification Kit (Qiagen). PCR amplification was used to enrich the collected fragments, followed by 2% agarose gel electrophoresis to recover the target fragments. Finally, the library was validated as follows: including: the Agilent 2100 bio analyzer instrument (Agilent DNA 1000 Reagents) was used to determine the average molecule length and RT-qPCR (TaqMan Probe) was used to quantify the libraries. Then, the qualified libraries were amplified on cBot to generate a cluster on the flow cells (PE Cluster Ki,Illumina) and the amplified flow cell were pair-end sequenced in individual lanes of the Illumina HiSeq4000 NGS platform (BGI-Shenzhen, ShenZhen, China).

### SNP discovery and genotyping

After filtering and splitting, Illumina raw sequence reads were retained. Briefly, low-quality data were discarded including: reads with adaptors, reads with more than 50% bases whose quality values ≤5, and reads that could not be identified with barcode sequences, which are used to classify reads between samples. Clean reads were mapped against the peach genome sequences from the GDR website (https://www.rosaceae.org/species/prunus_persica/genome_v2.0.a1) with SOAPaligner [61]. Finally, the SNP loci were identified from alignment results using soapsnp software (version 2.23) [62]. Genotypes of offspring individual were based on parental genotypes and markers were tested by chi-squared with the *P*-value set to 0.01. In addition, the family type was cross pollinators, which is a cross between two heterozygous diploid parents, and its linkage phases were originally unknown [63]. Three segregation types were genotyped, lmxll, nnxnp and hkxhk, with an expected segregation ratio for marker codes lmxll and, nnxnp was being 1:1, and that for lmxll, nnxnp and hkxhk was being 1:2:1. SNP markers with < 10% missing data in the each individual and consistent with the above standards were used for linkage map construction.

### Linkage mapping and QTL analysis

Construction of the genetic linkage map was accomplished using JoinMap (version 4.1) [63] with 6150 markers and cross pollinators family type. Initially, LGs were constructed using a LOD threshold of 3.0 to 4.0, Kosambi as the mapping function [64], and regression mapping as mapping algorithm. MapChart software (version 2.2) was used to make the map figures [65]. Mean phenotypic data from all 105 individuals (two parents and 103 F_1_ progeny) are list in Additional file 1. IciMapping software (version 4.1) was used to calculate the QTLs [66] with the ICIM-ADD method. LOD significance thresholds (*P* < 0.05) were analyzed by running 1000 permutation tests.

### Candidate gene mining in silico and a functional analysis

Mapping-associated markers were used to identify the homologous regions of QTLs on the physical map. Corresponding genes in QTLs were referred to the peach genome from GDR [42]. To identify the main biological functions of corresponding genes in QTLs, these genes were mapped to each node of the GO database (http://www.geneontology.org/) [67], and the pathway enrichment was also analyzed in KEGG (http://www.genome.jp/kegg/pathway.html).

### RNA isolation and expression analyses

The SH cultivars ‘YM’ and ‘XC’, and M cultivars ‘HJML’ and ‘XH8’ were used to expression analyses. The fruit samples were same as description from Guo et al. [59]. All fruit samples reached commercial maturity, had no diseases and mechanical damage with uniform maturity were randomly collected. And the fruits were stored in a room at at 25 ± 0.5 °C with a relative humidity of 75–85%. For HJML, YM and XH8 fruit samples were taken at 0, 4, 8 and 12 d postharvest; and for XC at 0, 4, 9 and 12d. The pulp from ten fruit were frozen in liquid nitrogen and stored at − 80 °C until further analysis. Three independent biological replicates were conducted for expression analysis. Total RNA was extracted from peach fruit samples using the RNAprep Pure Plant Kit (Polysaccharides & Polyphenolics-rich) (TIANGEN, Beijing, China), and an ultraviolet spectrophotometer (Eppendorf, Hamburg, Germany) and 1% agarose gels electrophoresis were used to detect RNA integrity and quality, respectively. cDNA was synthesized using the PrimeScript™ RT reagent Kit with gDNA Eraser (TaKaRa, Dalian, China). The NCBI/Primer-BLAST on-line server was used to design specific primers for each gene. All primer sequences are listed in Additional file 8. The *translation elongation factor 2* was used as the internal reference gene as in Tong et al. [68]. RT-qPCR was performed on a 7500 Real Time PCR System (Applied Biosystems, NY, USA) with SYBR®Premix Ex Taq™ (TaKaRa) and gene-specific primers in a volume of 20 μL. PCR conditions were as follows: an initial denaturation at 95 °C for 30 s, and 40 cycles of 95 °C for 5 s and 60 °C for 34 s. The specificity of primer amplifications was checked by a melting curve analysis. The comparative cycle threshold method (ΔΔCt) was used to analyze relative expression level data [69]. Each sample was analyzed in triplicate.

### Statistical analyses

Microsoft Excel 2010 was used to calculate standard errors (SEs). Graphs were produced using Origin 8.0 software. Significant differences between means of experimental data and a correlation analysis were determined using SPSS 19.0 software (SPSS, Chicago, IL, USA).

## Additional files

Additional file 1: Phenotypic identification of the 103 F_1_ individuals from a cross between two peach cultivars the SH-type cultivar ‘YM’ and the M-type cultivar ‘HJML’. a. Identification of SH phenotype based on the ethylene production in 2016; b. Identification of SH phenotype based on the fruit firmness in 2017; c. Phenotype identification used for QTL analysis. (DOCX 16 kb)

Additional file 2: Statistics for the data of RAD-seq in the parent plants and those of their individual offspring. (XLSX 21 kb)

Additional file 3: 6150 SNP markers identified after filtering used to construct the linkage map in peach. (XLSX 2185 kb)

Additional file 4: Analysis of the SNP polymorphic sites in the parent plants and their individual offspring in peach. (XLSX 21652 kb)

Additional file 5: Numbers of SNP transitions types in parent plants and their offspring in peach. (DOCX 21 kb)

Additional file 6: Discrete genes obtained from QTL regions and the alignment results to GO database and KEGG pathway database. (XLSX 23 kb)

Additional file 7: Scatter plot illustrating pathway rich factor analysis. (JPG 3748 kb)

Additional file 8 Specific primers used for RT-qPCR to detect the expression of 11 candidate genes. (DOCX 14 kb)

## Abbreviations

M: Melting
NM: No-melting
SH: Stony hard
ABA: Abscisic acid
NGS: Next-generation sequencing
RAD-seq: Restriction-site associated DNA sequencing
QTL: Quantitative trait loci
LG: Linkage group
SE: Standard error
GDR: Genome Database for *Rosaceae*
SNP: Single nucleotide polymorphism
YM: Yumyeoung
HJML: Hu Jing Mi Lu
XH8: Xia Hui 8
XC: Xia Cui
LOD: Likelihood of odd
RT-qPCR: real-time quantitative PCR
GO: Gene Ontology
KEGG: Kyoto Encyclopedia of Genes and Genomes
