# Supplementary material for: High-density genetic map construction and quantitative trait loci analysis of the stony hard phenotype in peach based on restriction-site associated DNA sequencing
Source: BMC Genomics. 2018 Aug 14;19:612. doi: 10.1186/s12864-018-4952-y (PMC6092793; doi:10.1186/s12864-018-4952-y)
Supplement: Supplementary file 1 — Phenotypic identification of the 103 F1 individuals from a cross between two peach cultivars the SH-type cultivar ‘YM’ and the M-type cultivar ‘HJML’. a. Identification of SH phenotype based on the ethylene production in 2016; b. Identification of SH phenotype based on the fruit firmness in 2017; c. Phenotype identification used for QTL analysis. (DOCX 16 kb) [file 12864_2018_4952_MOESM1_ESM.docx]

Additional file 1: Phenotypic identification of the 103 F_1_ individuals from a cross between two peach cultivars the SH-type cultivar ‘YM’ and the M-type cultivar ‘HJML’

a. Identification of SH phenotype based on the ethylene production in 2016

| SH phenotype | M phenotype | Unidentified phenotype |
| --- | --- | --- |
| 3,9,11,12,13,14,15,17,28,30,34,35,41,44,45,49,50,51,53,55,58,60,61,62,64,  66,68,73,74,75,79,81,82,83,85,86,90,  92,93,99,100,101 | 1,2,4,6,7,8,10,16,18,19,20,21,22,23,24,25,26,27,29,31,32,33,36,38,40,  43,46,47,48,52,54,56,57,59, 65,72,  76,77,78,80,84,87,89,91,94,95,96,  97,98 | 5,37,39,42,63,67,  69,70,71,88,102,  103 |

Note: Each number represent the single F_1_ offspring

b. Identification of SH phenotype based on the fruit firmness in 2017

| SH phenotype | M phenotype | Unidentified phenotype |
| --- | --- | --- |
| 3,5,9,11,12,13,14,15,17,28,30,34,35,37,41,44,45,49,50,51,53,55,58,60,61,62,64,66,68,70,74,75,79,81,82,83,85,86,88,90,92,99,100,101,103, | 1,2,4,6,7,8,10,16,18,19,20,21,22,23,24,25,26,27,29,31,32,33,36,38,40,43,46,47,48,52,54,56,63,65,67,72,77, 80,84,87,89,91,94, 96,97,98,102, | 39,42,57,59,69,71,73,76,78, 93,95 |

Note: Each number represent the single F_1_ offspring

c.Phenotype identification used for QTL analysis

| SH phenotype | M phenotype | Total number |
| --- | --- | --- |
| 3,9,11,12,13,14,15,17,28,30,34,35,41,44,45,49,50,51,53,55,58,60,61,62,64,  66,68,73,74,75,79,81,82,83,85,86,90,  92,93,99,100,101 | 1,2,4,6,7,8,10,16,18,19,20,21,22,23,24,25,26,27,29,31,32,33,36,38,40,  43,46,47,48,52,54,56,57,59, 65,72,  76,77,78,80,84,87,89,91,94,95,96,  97,98 | 91 |

Note: Each number represent the single F1 offspring
