# Supplementary material for: High-density genetic map construction and quantitative trait loci analysis of the stony hard phenotype in peach based on restriction-site associated DNA sequencing
Source: BMC Genomics. 2018 Aug 14;19:612. doi: 10.1186/s12864-018-4952-y (PMC6092793; doi:10.1186/s12864-018-4952-y)
Supplement: Supplementary file 5 — Numbers of SNP transitions types in parent plants and their offspring in peach. (DOCX 21 kb) [file 12864_2018_4952_MOESM5_ESM.docx]

Additional file 5: Numbers of SNP transitions types in parent plants and their offspring in peach.

| sample | Numbers of SNP transitions types | | | | | |
| --- | --- | --- | --- | --- | --- | --- |
|  | A/T | A/G | A/C | G/T | G/C | C/T |
| 1 | 2299 | 7644 | 2242 | 2274 | 1677 | 7521 |
| 2 | 2379 | 8061 | 2333 | 2401 | 1698 | 7971 |
| 3 | 2457 | 7867 | 2321 | 2490 | 1739 | 7870 |
| 4 | 2543 | 8397 | 2455 | 2569 | 1813 | 8353 |
| 5 | 2801 | 9456 | 2706 | 2841 | 1999 | 9404 |
| 6 | 2206 | 7208 | 2149 | 2277 | 1603 | 7201 |
| 7 | 2152 | 7025 | 2040 | 2198 | 1557 | 6990 |
| 8 | 2488 | 8206 | 2379 | 2481 | 1765 | 8203 |
| 9 | 2898 | 9137 | 2705 | 2825 | 1970 | 9073 |
| 10 | 2816 | 8712 | 2549 | 2670 | 1872 | 8678 |
| 11 | 2605 | 8209 | 2374 | 2477 | 1764 | 8148 |
| 12 | 2700 | 8934 | 2565 | 2618 | 1906 | 8888 |
| 13 | 2297 | 7293 | 2037 | 2196 | 1553 | 7309 |
| 14 | 2672 | 8456 | 2445 | 2564 | 1810 | 8419 |
| 15 | 2603 | 8408 | 2412 | 2489 | 1746 | 8233 |
| 16 | 2969 | 9224 | 2723 | 2821 | 1989 | 9200 |
| 17 | 2896 | 9376 | 2715 | 2783 | 1987 | 9408 |
| 18 | 2113 | 6882 | 1979 | 2090 | 1423 | 6828 |
| 19 | 3009 | 9927 | 2804 | 2937 | 2072 | 9891 |
| 20 | 2512 | 7910 | 2359 | 2395 | 1726 | 7893 |
| 21 | 2848 | 9262 | 2654 | 2766 | 1950 | 9007 |
| 22 | 2511 | 8190 | 2381 | 2480 | 1780 | 8127 |
| 23 | 2402 | 7343 | 2142 | 2254 | 1610 | 7339 |
| 24 | 2589 | 8348 | 2422 | 2506 | 1810 | 8190 |
| 25 | 2413 | 7704 | 2257 | 2318 | 1576 | 7570 |
| 26 | 2501 | 8196 | 2360 | 2421 | 1718 | 8008 |
| 27 | 2453 | 8184 | 2337 | 2446 | 1700 | 8130 |
| 28 | 2801 | 8880 | 2605 | 2695 | 1880 | 8880 |
| 29 | 1890 | 6121 | 1757 | 1853 | 1349 | 6091 |
| 30 | 2162 | 6877 | 2025 | 2063 | 1505 | 6857 |
| 31 | 2516 | 7963 | 2348 | 2476 | 1719 | 8028 |
| 32 | 2652 | 8591 | 2482 | 2588 | 1775 | 8520 |
| 33 | 2354 | 7438 | 2122 | 2232 | 1574 | 7355 |
| 34 | 2865 | 9324 | 2687 | 2777 | 1919 | 9177 |
| 35 | 2773 | 8954 | 2561 | 2722 | 1961 | 8838 |
| 36 | 2754 | 8960 | 2588 | 2709 | 1919 | 8957 |
| 37 | 2882 | 9282 | 2706 | 2751 | 1973 | 9278 |
| 38 | 2777 | 8933 | 2591 | 2730 | 1894 | 8664 |
| 39 | 2946 | 9171 | 2704 | 2795 | 1954 | 9155 |
| 40 | 2346 | 7408 | 2229 | 2259 | 1588 | 7428 |
| 41 | 2697 | 8446 | 2501 | 2613 | 1825 | 8514 |
| 42 | 3084 | 10121 | 2923 | 2991 | 2095 | 10098 |
| 43 | 2744 | 8732 | 2595 | 2609 | 1842 | 8652 |
| 44 | 2944 | 9347 | 2739 | 2831 | 1970 | 9225 |
| 45 | 3008 | 9590 | 2755 | 2866 | 2015 | 9469 |
| 46 | 2446 | 7814 | 2229 | 2372 | 1689 | 7744 |
| 47 | 2856 | 9559 | 2739 | 2800 | 2024 | 9412 |
| 48 | 2512 | 7860 | 2339 | 2436 | 1709 | 7799 |
| 49 | 2453 | 7487 | 2191 | 2264 | 1629 | 7516 |
| 50 | 2830 | 9098 | 2660 | 2706 | 1934 | 8941 |
| 51 | 2676 | 8617 | 2471 | 2604 | 1886 | 8593 |
| 52 | 2871 | 9252 | 2686 | 2803 | 2017 | 9138 |
| 53 | 2678 | 8489 | 2492 | 2556 | 1793 | 8558 |
| 54 | 2767 | 8814 | 2541 | 2649 | 1887 | 8744 |
| 55 | 2741 | 8726 | 2567 | 2697 | 1869 | 8661 |
| 56 | 2435 | 7949 | 2308 | 2426 | 1695 | 7820 |
| 57 | 2445 | 8025 | 2354 | 2420 | 1751 | 7986 |
| 58 | 2625 | 8263 | 2447 | 2510 | 1786 | 8220 |
| 59 | 2487 | 7871 | 2308 | 2382 | 1686 | 7700 |
| 60 | 2605 | 8380 | 2450 | 2483 | 1852 | 8365 |
| 61 | 2793 | 8890 | 2653 | 2725 | 1909 | 8832 |
| 62 | 2565 | 8143 | 2387 | 2448 | 1741 | 8214 |
| 63 | 2650 | 8411 | 2427 | 2508 | 1810 | 8362 |
| 64 | 2817 | 8783 | 2564 | 2661 | 1861 | 8622 |
| 65 | 2630 | 8334 | 2433 | 2547 | 1813 | 8271 |
| 66 | 2228 | 7035 | 2069 | 2186 | 1608 | 7131 |
| 67 | 2505 | 7844 | 2258 | 2402 | 1688 | 7895 |
| 68 | 2885 | 9155 | 2636 | 2781 | 1995 | 9217 |
| 69 | 2715 | 8804 | 2574 | 2635 | 1862 | 8651 |
| 70 | 2653 | 8663 | 2515 | 2574 | 1813 | 8530 |
| 71 | 2890 | 8957 | 2593 | 2717 | 1870 | 8951 |
| 72 | 2780 | 9137 | 2662 | 2740 | 1940 | 9086 |
| 73 | 2864 | 9266 | 2680 | 2786 | 1983 | 9265 |
| 74 | 2867 | 9027 | 2623 | 2761 | 1891 | 8933 |
| 75 | 2693 | 8261 | 2466 | 2553 | 1765 | 8266 |
| 76 | 2763 | 8715 | 2583 | 2634 | 1887 | 8686 |
| 77 | 2492 | 8094 | 2385 | 2469 | 1722 | 8150 |
| 78 | 2005 | 6423 | 1858 | 1927 | 1407 | 6492 |
| 79 | 2530 | 8113 | 2349 | 2470 | 1784 | 8155 |
| 80 | 2440 | 7747 | 2290 | 2371 | 1701 | 7742 |
| 81 | 2428 | 8234 | 2365 | 2466 | 1764 | 8211 |
| 82 | 2671 | 8743 | 2542 | 2629 | 1895 | 8766 |
| 83 | 2655 | 8817 | 2549 | 2646 | 1893 | 8684 |
| 84 | 2498 | 8340 | 2384 | 2506 | 1834 | 8342 |
| 85 | 2757 | 9057 | 2668 | 2757 | 1906 | 9024 |
| 86 | 2599 | 8432 | 2471 | 2554 | 1839 | 8284 |
| 87 | 2759 | 8920 | 2629 | 2699 | 1967 | 8945 |
| 88 | 2978 | 9652 | 2812 | 2917 | 2099 | 9539 |
| 89 | 2298 | 7208 | 2082 | 2197 | 1557 | 7264 |
| 90 | 2424 | 7678 | 2188 | 2333 | 1624 | 7727 |
| 91 | 2312 | 7095 | 2123 | 2250 | 1527 | 7148 |
| 92 | 2060 | 6838 | 2002 | 2041 | 1452 | 6720 |
| 93 | 2164 | 6924 | 2003 | 2082 | 1490 | 6897 |
| 94 | 2502 | 7904 | 2333 | 2394 | 1679 | 7882 |
| 95 | 2584 | 8196 | 2359 | 2433 | 1723 | 8091 |
| 96 | 2197 | 6937 | 2021 | 2080 | 1476 | 6889 |
| 97 | 1798 | 5752 | 1739 | 1759 | 1268 | 5838 |
| 98 | 2447 | 7953 | 2340 | 2405 | 1657 | 7982 |
| 99 | 2956 | 9358 | 2752 | 2862 | 1994 | 9304 |
| 100 | 2439 | 7780 | 2255 | 2266 | 1640 | 7652 |
| 101 | 2621 | 8382 | 2464 | 2606 | 1822 | 8335 |
| 102 | 2165 | 7088 | 2073 | 2129 | 1523 | 6990 |
| 103 | 2344 | 7689 | 2213 | 2252 | 1689 | 7680 |
| HJML | 3674 | 11604 | 3421 | 3554 | 2480 | 11438 |
| YM | 4376 | 12773 | 3955 | 3999 | 2680 | 12768 |

Note: A/T, A/G, A/C, G/T, G/C and C/T represent the different SNP transitions types.
