# Supplementary figures and images for: High-density genetic map construction and quantitative trait loci analysis of the stony hard phenotype in peach based on restriction-site associated DNA sequencing
Source: BMC Genomics. 2018 Aug 14;19:612. doi: 10.1186/s12864-018-4952-y (PMC6092793; doi:10.1186/s12864-018-4952-y)

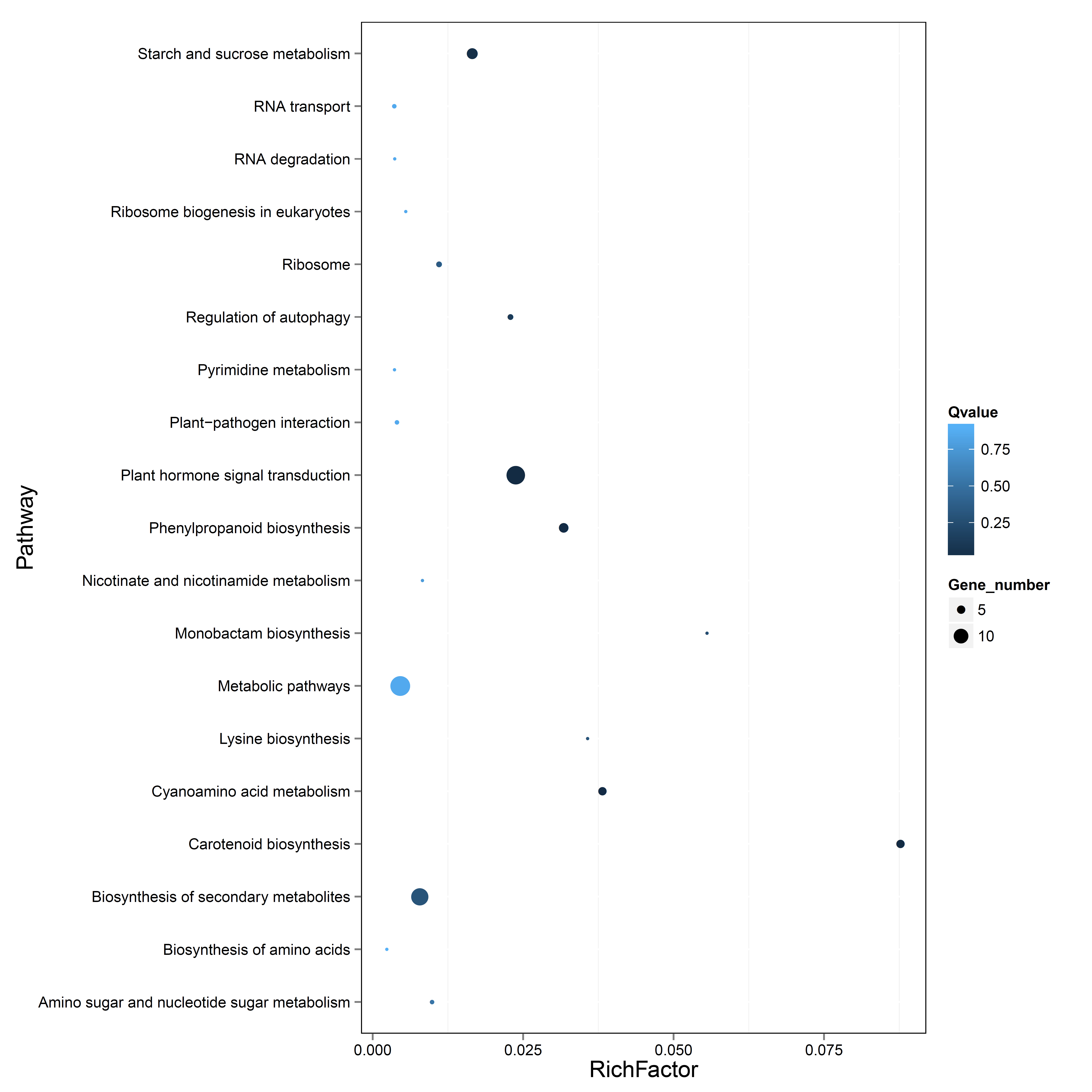

Supplement: Supplementary file 7 — Scatter plot illustrating pathway rich factor analysis. (JPG 3748 kb) [file 12864_2018_4952_MOESM7_ESM.jpg]
