# Supplementary material for: High-density genetic map construction and quantitative trait loci analysis of the stony hard phenotype in peach based on restriction-site associated DNA sequencing
Source: BMC Genomics. 2018 Aug 14;19:612. doi: 10.1186/s12864-018-4952-y (PMC6092793; doi:10.1186/s12864-018-4952-y)
Supplement: Supplementary file 8 — Specific primers used for RT-qPCR to detect the expression of 11 candidate genes. (DOCX 14 kb) [file 12864_2018_4952_MOESM8_ESM.docx]

Additional file 8: Specific primers used for RT-qPCR to detect the expression of 11 candidate genes

| Gene Name | Primers (5’ to 3’) forward | Primers(5’ to 3’) reverse |
| --- | --- | --- |
| *Prupe.6G147600.1* | GACCAACCAAGCATCTCT | CTATGGCTAATGTCAGGATTAC |
| *Prupe.6G150800.2* | GGAACTGAAGGACAAGGT | GGTTGGCTTAGTAGTGAGA |
| *Prupe.6G156500.1* | GCTTCCTCCTCTGCTTAT | ACTCATCACACGCTACAT |
| *Prupe.6G150900.1* | AATGTGATGGTAGTGGAGAT | GATGCCTGTTGGATAAGC |
| *Prupe.6G156000.1* | AGAACTTATGGACTGCTGAA | TACCTCATCAACCGTCTTC |
| *Prupe.6G151800.1* | CTTGGCTAAGGCATTGTC | GTGGTTGTTGGAAGAATAGG |
| *Prupe.6G154900.1* | ACTTGAAGAGCCTCGTTA | CCACTGGTAGGAATTGTTC |
| *Prupe.6G156700.1* | GAGCAATGGATGATGAGAG | CTGTATGGAGGCACTGTAA |
| *Prupe.6G157900.2* | GACGATAATCCAAGGTGTAAC | TCCAATCAATGTGCCAAC |
| *Prupe.6G158300.1* | TTCTGGCTCTGCTTCTTAT | CTGGTGTCTGGAGTATCTAC |
| *Prupe.6G158400.1* | CTGGAGGATTGTGACTGT | AAGCACCATTACCAATAGAG |
| *TEF2* | GGTGTGACGATGAAGAGTGATG | TGAAGGAGAGGGAAGGTGAAAG |
